# *Ochraceocephala foeniculi* gen. et sp. nov., a new pathogen causing crown rot of fennel in Italy

**DOI:** 10.3897/mycokeys.66.48389

**Published:** 2020-03-30

**Authors:** Dalia Aiello, Alessandro Vitale, Giancarlo Polizzi, Hermann Voglmayr

**Affiliations:** 1 Dipartimento di Agricoltura, Alimentazione e Ambiente, sezione Patologia Vegetale, University of Catania, Via S. Sofia 100, 95123 Catania, Italy University of Catania Catania Italy; 2 Institute of Forest Entomology, Forest Pathology and Forest Protection, Department of Forest and Soil Sciences, BOKU - University of Natural Resources and Life Sciences, Franz Schwackhöfer Haus, Peter-Jordan-Straße 82/I, 1190 Vienna, Austria University of Natural Resources and Life Sciences Vienna Austria; 3 Division of Systematic and Evolutionary Botany, Department of Botany and Biodiversity Research, University of Vienna, Rennweg 14, 1030 Wien, Austria University of Vienna Vienna Austria

**Keywords:** Fungal disease, Leptosphaeriaceae, pathogenicity, susceptibility

## Abstract

A new disease of fennel is described from Sicily (southern Italy). Surveys of the disease and sampling were conducted during spring 2017 and 2018 in Adrano and Bronte municipalities (Catania province) where this crop is widely cultivated. Isolations from the margin of symptomatic tissues resulted in fungal colonies with the same morphology. Pathogenicity tests with one isolate of the fungus on 6-month-old plants of fennel reproduced similar symptoms to those observed in nature. Inoculation experiments to assess the susceptibility of six different fennel cultivars to infection by the pathogen showed that the cultivars ‘Narciso’, ‘Apollo’, and ‘Pompeo’ were more susceptible than ‘Aurelio’, ‘Archimede’, and ‘Pegaso’. Phylogenetic analyses based on a matrix of the internal transcribed spacer (ITS), the large subunit (LSU), and the small subunit (SSU) rDNA regions revealed that the isolates represent a new genus and species within the Leptosphaeriaceae, which is here described as *Ochraceocephala foeniculi* gen. et sp. nov. This study improves the understanding of this new fennel disease, but further studies are needed for planning effective disease management strategies. According to the results of the phylogenetic analyses, *Subplenodomus iridicola* is transferred to the genus *Alloleptosphaeria* and *Acicuseptoria rumicis* to *Paraleptosphaeria*.

## Introduction

Fennel (*Foeniculum vulgare* Mill.), native in arid and semi-arid regions of southern Europe and the Mediterranean area, is used as a vegetable, herb, and seed spice in the food, pharmaceutical, cosmetic, and healthcare industries. Italy is the leading world producer of fennel (around 85% of the world production), with 20,035 ha of area cultivated and a total production of 537,444 tons. Fennel represents an important crop widely cultivated in Sicily (southern Italy) with 1,620 ha harvested and a production of 35,930 tons (ISTAT 2018). Several diseases caused by fungi have been reported from this crop throughout the world (Table 1). Amongst soilborne diseases, brown rot and wilt caused by *Phytophthora megasperma* and crown rot caused by *Didymella glomerata* (syn. *Phoma glomerata*) were reported in Italy (Cacciola et al. 2006; Lahoz et al. 2007).

In 2017, a new disease was first observed on fennel in a farm of Adrano area (Catania province, eastern Sicily, Italy). The disease symptoms were necrotic lesions on the crown, root, and stem of fennel plants. Disease incidence initially was about 5% on ‘Apollo’ cultivar. However, in 2018 different surveys conducted in the same area showed a high increase of the incidence on three different cultivars with yield losses of about 20–30%. The aims of the present study were to identify the causal agent obtained from symptomatic fennel plants, using morphological characteristics and DNA sequence analyses, to evaluate the pathogenicity of one representative isolate and to evaluate the susceptibility of different cultivars of fennel to the newly described disease.

**Table 1.** Main diseases caused by fungal pathogens on fennel.

**Disease**	**Fungal pathogen**	**Reference**
Collar rot	*Sclerotium rolfsii*	Khare et al. 2014
Damping off and Root rot	*Pythium* spp.	Khare et al. 2014; Koike et al. 2015
Vascular wilt	*Fusarium oxysporum*	Shaker and Alhamadany 2015
Vascular wilt	*Verticillium dahliae*	Ghoneem et al. 2009
Root and Foot rot	*Rhizoctonia solani*	Shaker and Alhamadany 2015
Brown rot and Wilt	*Phytophthora megasperma*	Cacciola et al. 2006
Stem rot	*Sclerotinia sclerotiorum*	Choi et al. 2016
Blight and Leaf spot	*Alternaria alternata*	D'Amico et al. 2008
Blight and Leaf spot	*Ascochyta foeniculina*	Khare et al. 2014
Blight and Leaf spot	*Fusoidiella anethi*	Taubenrauch et al. 2008
	syn. *Cercospora foeniculi*	
	*Cercosporidium punctum*	
	*Mycosphaerella anethi*	
	*M. foeniculi*	
	*Passalora kirchneri*	
	*P. puncta*	
	*Ramularia foeniculi*	
Umbel browning and Stem necrosis	*Diaporthe angelicae*	Rodeva and Gabler 2011
Downy mildew	*Plasmopara mei-foeniculi*	Khare et al. 2014
	syn. *P. nivea* sensu lato	
Powdery mildew	*Leveillula languinosa*	Khare et al. 2014
Powdery mildew	*Erysiphe heraclei*	Choi et al. 2015
Leaf spot	*Leptosphaeria purpurea*	Odstrčilová et al. 2002
Leaf spot	*Subplenodomus apiicola*	Odstrčilová et al. 2002
	syn. *Phoma apiicola*	
Leaf spot and blight	*Phoma herbarum*	Shaker and Alhamadany 2015
Crown rot	*Didymella glomerata*	Lahoz et al. 2007
	syn. *Phoma glomerata*	

## Materials and methods

### Collection of samples and fungal isolates

In order to identify the causal agent of the fennel disease, 30 samples were collected during several surveys in Adrano and Bronte area (Catania province, eastern Sicily). Pieces of tissue obtained from different parts of fennel plants (crown, root, and stem) were surface disinfected for 1 min in 1.5% sodium hypochlorite solution, rinsed in sterile water, placed on potato dextrose agar (3.9% PDA, Oxoid, Basingstoke, UK) amended with 100 mg/L of streptomycin sulfate (Sigma-Aldrich, USA) to prevent bacteria growth, and then incubated at 25 ± 1 °C for seven days. Fungal colonies consistently grown from symptomatic tissues were subcultured on new PDA plates. Subsequently, single-spore isolates were obtained from these pure cultures and stored at –20 °C in sterile 15% glycerol solution. The fungal isolates were provisionally identified by cultural and morphological characteristics, and they were deposited in the culture collection of the Department of Agriculture, Food and Environment, University of Catania. One representative isolate (Di3A-F1; ex holotype culture) was deposited at the Westerdijk Fungal Biodiversity Institute (**CBS**), Utrecht, the Netherlands. The holotype specimen of the new pathogen species was deposited in the fungarium of the Department of Botany and Biodiversity Research, University of Vienna (**WU**).

### Morphology

For culture characteristics, cultures were grown on 2% (w/v) malt extract agar (MEA, VWR) and on corn meal agar (CMA, Sigma-Aldrich) supplemented with 2% w/v dextrose (CMD). Colony diameters and morphologies were determined after seven days of incubation at room temperature (22 ± 1 °C) and daylight.

Microscopic observations were made in tap water. Methods of microscopy included stereomicroscopy using a Nikon SMZ 1500 equipped with a Nikon DS-U2 digital camera, and Nomarski differential interference contrast (DIC) using a Zeiss Axio Imager.A1 compound microscope equipped with a Zeiss Axiocam 506 colour digital camera. Images and data were gathered using the NIS-Elements D v. 3.22.15 or Zeiss ZEN Blue Edition software packages. Measurements are reported as maxima and minima in parentheses and the range representing the mean plus and minus the standard deviation of a number of measurements given in parentheses.

### DNA extraction and PCR amplification

The extraction of genomic DNA from pure cultures was performed by using the Wizard Genomic DNA Purification Kit (Promega Corporation, WI, USA). Partial regions of six loci (ITS, LSU, and SSU rDNA, *RPB2*, *TEF1*, *TUB2*) were amplified; for details on the primers and annealing temperatures used for PCR and sequencing, see Table 2. The PCR products were sequenced in both directions by Macrogen Inc. (South Korea) or at the Department of Botany and Biodiversity Research, University of Vienna using the ABI PRISM Big Dye Terminator Cycle Sequencing Ready Reaction Kit v. 3.1 (Applied Biosystems, Warrington, UK) and an automated DNA sequencer (3730xl Genetic Analyser, Applied Biosystems). The DNA sequences generated were assembled with Lasergene SeqMan Pro (DNASTAR, Madison, USA). Sequences generated during the present study were uploaded to Genbank (Table 3).

**Table 2.** Primers used to amplify and sequence the nuclear internal transcribed spacer (ITS), large subunit (LSU) and small subunit (SSU) rDNA regions, the RNA polymerase II second largest subunit (*RPB2*) gene, the translation elongation factor 1-α (*TEF1*) gene and the β-tubulin (*TUB2*) gene.

**Gene**	**Primer**	**Sequence (5'–3')**	**Direction**	**Annealing t (°C)**	**Reference**
ITS	ITS5	GGAAGTAAAAGTCGTAACAAGG	forward	48	White et al. 1990
	ITS4	TCCTCCGCTTATTGATATGC	reverse		White et al. 1990
LSU	LR0R	GTACCCGCTGAACTTAAGC	forward	48	Vilgalys and Hester 1990
	LR5	TCCTGAGGGAAACTTCG	reverse		Vilgalys and Hester 1990
ITS-LSU	V9G	TTAAGTCCCTGCCCTTTGTA	forward	55	Hoog and Gerrits van den Ende 1998
	LR5	TACTTGAAGGAACCCTTACC	reverse		Vilgalys and Hester 1990
	LR2R-A^z^	CAGAGACCGATAGCGCAC	forward		Voglmayr et al. 2012
	LR3^z^	CCGTGTTTCAAGACGGG	reverse		Vilgalys and Hester 1990
	ITS4^z^	TCCTCCGCTTATTGATATGC	reverse		White et al. 1990
SSU	NS1	GTAGTCATATGCTTGTCTC	forward	48	White et al. 1990
	NS4	CTTCCGTCAATTCCTTTAAG	reverse		White et al. 1990
*RPB2*	RPB2-5F2	GGGGWGAYCAGAAGAAGGC	forward	52	Sung et al. 2007
	RPB2-7cR	CCCATRGCTTGYTTRCCCAT	reverse		Liu et al. 1999
*TEF1*	EF1-728F	CATCGAGAAGTTCGAGAAGG	forward	52	Carbone and Kohn 1999
	EF1-986R	TACTTGAAGGAACCCTTACC	reverse		Carbone and Kohn 1999
	EF1-728F	CATCGAGAAGTTCGAGAAGG	forward	55	Carbone and Kohn 1999
	TEF1-LLErev	AACTTGCAGGCAATGTGG	reverse		Jaklitsch et al. 2005
	TEF1_INTF^z^	CCGTGAYTTCATCAAGAACATG	forward		Jaklitsch 2009
	TEF1_INT2^z^	CCACTTNGTNGTGTCCATCTTRTT	reverse		Voglmayr and Jaklitsch 2017
*TUB2*	T1	AACATGCGTGAGATTGTAAGT	forward	52	O'Donnell and Cigelnik 1997
	bt2b	ACCCTCAGTGTAGTGACCCTTGGC	reverse		Glass and Donaldson 1995

^z^ internal primers used only for sequencing

**Table 3.** Characteristics and accession numbers of isolates collected from fennel plants in Sicily.

**Strain^1^**	**Year**	**Cultivar**	**Farm**	**ITS^2^**	**LSU^2^**	**SSU^2^**	***RPB2*^2^**	***TEF1*^2^**	***TUB2*^2^**
Di3AF1 = CBS 145654*	2017	Apollo	Farm 1	MN516753	MN516774	MN516743	MN520145	MN520149	MN520147
**Di3AF2**	2017	Apollo	Farm 1	MN516754	MN516775	MN516744			
**Di3AF3**	2018	Apollo	Farm 1	MN516755	MN516776	MN516745			
Di3AF4	2018	Apollo	Farm 1						
**Di3AF5**	2018	Apollo	Farm 1	MN516756	MN516777	MN516746			
**Di3AF6**	2018	Apollo	Farm 1	MN516757	MN516778	MN516747			
**Di3AF7**	2018	Apollo	Farm 1	MN516758					
**Di3AF8**	2018	Apollo	Farm 1	MN516759					
**Di3AF9**	2018	Apollo	Farm 1	MN516760	MN516779	MN516748			
**Di3AF10**	2018	Apollo	Farm 1	MN516761	MN516780	MN516749	MN520146	MN520150	MN520148
**Di3AF11**	2018	Apollo	Farm 1	MN516762					
**Di3AF12**	2018	Apollo	Farm 1	MN516763					
**Di3AF13**	2018	Apollo	Farm 1	MN516764	MN516781	MN516750			
**Di3AF14**	2018	Apollo	Farm 1	MN516765	MN516782	MN516751			
**Di3AF15**	2018	Apollo	Farm 1	MN516766	MN516783	MN516752			
**Di3AF16**	2018	Apollo	Farm 1	MN516767					
**Di3AF17**	2018	Apollo	Farm 1	MN516768					
Di3AF18	2018	Narciso	Farm 2						
**Di3AF19**	2018	Narciso	Farm 2	MN516769					
**Di3AF20**	2018	Narciso	Farm 2	MN516770					
**Di3AF21**	2018	Narciso	Farm 2	MN516771					
Di3AF22	2018	Narciso	Farm 2						
Di3AF23	2018	Narciso	Farm 2						
Di3AF24	2018	Narciso	Farm 2						
Di3AF25	2018	Narciso	Farm 2						
Di3AF26	2018	Narciso	Farm 3						
Di3AF27	2018	Narciso	Farm 3						
Di3AF28	2018	Narciso	Farm 3						
Di3AF29	2018	Narciso	Farm 4						
**Di3AF30**	2018	Narciso	Farm 4	MN516772					
Di3AF31	2018	Narciso	Farm 4						
**Di3AF32**	2018	Aurelio	Farm 5	MN516773					

**Di3A**: Cultures stored at the University of Catania, Italy; **CBS**: Culture collection of the Westerdijk Fungal Biodiversity Institute, Utrecht, The Netherlands. Isolates in bold were sequenced in the present study. ^2^**ITS**: internal transcribed spacer rDNA region, **LSU**: large subunit rDNA region, **SSU**: small subunit rDNA region, ***RPB2***: RNA polymerase II second largest subunit gene, ***TEF1***: translation elongation factor 1-α, ***TUB2***: β-tubulin gene.*Ex-type strain.

### Phylogenetic analysis

According to the results of BLAST searches in GenBank, the newly generated ITS, LSU, and SSU rDNA sequences of the fennel pathogen were aligned with selected sequences of Leptosphaeriaceae from Gruyter et al. (2013) and complemented with a few recent additions from GenBank. The familial and generic concept of Leptosphaeriaceae implemented here follows the molecular phylogenetic studies of Gruyter et al. (2013), Ariyawansa et al. (2015), and Phookamsak et al. (2019). Due to insufficient *RPB2*, *TEF1*, and *TUB2* sequence data available in Genbank for the study group, the sequences of these markers could not be included in phylogenetic analyses, but they were deposited in GenBank (Table 3). A combined SSU-ITS-LSU rDNA matrix was produced for phylogenetic analyses, with six species of *Coniothyrium* (*C. carteri*, *C. dolichi*, *C. glycines*, *C. multiporum*, *C. telephii*, *C. palmarum*) from Coniothyriaceae added as the outgroup according to the results of the phylogenetic analyses of Gruyter et al. (2013). As the rDNA sequences of the fennel pathogen isolates were (almost) identical (see Results section below), only a single isolate (CBS 145654 = Di3A-F1; ex holotype strain) was included in the final matrix. The GenBank accession numbers of sequences used in the analyses are given in Table 4. Sequence alignments were produced with the server version of MAFFT (http://mafft.cbrc.jp/alignment/server), checked and refined using BioEdit v. 7.2.6 (Hall 1999). The combined data matrix contained 3312 characters; i.e. 607 nucleotides of the ITS, 1333 nucleotides of the LSU and 1372 nucleotides of the SSU).

**Table 4.** Isolates and accession numbers used in the phylogenetic analyses. Isolate/sequences in bold were isolated/sequenced in the present study.

Taxon	Culture, specimen	Host, substrate	Country	GenBank accession no		
				ITS	LSU	SSU
*Alloleptosphaeria iridicola*	CBS 143395	*Iris* sp. (Iridaceae)	United Kingdom	MH107919	MH107965	
*Alloleptosphaeria italica*	MFLUCC 14-934	*Clematis vitalba* (Ranunculaceae)	Italy	KT454722	KT454714	
*Alternariaster bidentis*	CBS 134021	*Bidens sulphurea* (Asteraceae)	Brazil	KC609333	KC609341	
*Alternariaster centaureae-diffusae*	MFLUCC 14-0992	*Centaurea diffusa* (Asteraceae)	Russia	KT454723	KT454715	KT454730
*Alternariaster helianthi*	CBS 119672	*Helianthus* sp. (Asteraceae)	USA	KC609337	KC584368	KC584626
*Alternariaster trigonosporus*	MFLU 15-2237	*Cirsium* sp. (Asteraceae)	Russia	KY674857	KY674858	
*Coniothyrium carteri*	CBS 105.91	*Quercus robur* (Fagaceae)	Germany	JF740181	GQ387594	GQ387533
*Coniothyrium dolichi*	CBS 124140	*Dolichos biforus* (Fabaceae)	India	JF740183	GQ387611	GQ387550
*Coniothyrium glycines*	CBS 124455	*Glycine max* (Fabaceae)	Zambia	JF740184	GQ387597	GQ387536
*Coniothyrium multiporum*	CBS 501.91	Unknown	Egypt	JF740186	GU238109	
*Coniothyrium palmarum*	CBS 400.71	*Chamaerops humilis* (Arecaceae)	Italy	AY720708	EU754153	EU754054
*Coniothyrium telephii*	CBS 188.71	Air	Finland	JF740188	GQ387599	GQ387538
*Heterosporicola chenopodii*	CBS 448.68	*Chenopodium album* (Chenopodiaceae)	Netherlands	FJ427023	EU754187	EU754088
*Heterosporicola dimorphospora*	CBS 165.78	*Chenopodium quinoa* (Chenopodiaceae)	Peru	JF740204	JF740281	JF740098
*Leptosphaeria conoidea*	CBS 616.75	*Lunaria annua* (Brassicaceae)	Netherlands	JF740201	JF740279	JF740099
*Leptosphaeria doliolum*	CBS 505.75	*Urtica dioica* (Urticaceae)	Netherlands	JF740205	GQ387576	GQ387515
*Leptosphaeria errabunda*	CBS 617.75	*Solidago* sp. (hybrid) (Asteraceae)	Netherlands	JF740216	JF740289	
*Leptosphaeria macrocapsa*	CBS 640.93	*Mercurialis perennis* (Euphorbiaceae)	Netherlands	JF740237	JF740304	
*Leptosphaeria pedicularis*	CBS 126582	*Gentiana punctata* (Gentianaceae)	Switzerland	JF740223	JF740293	
*Leptosphaeria sclerotioides*	CBS 144.84	*Medicago sativa* (Fabaceae)	Canada	JF740192	JF740269	
*Leptosphaeria slovacica*	CBS 389.80	*Balota nigra* (Lamiaceae)	Netherlands	JF740247	JF740315	JF740101
*Leptosphaeria sydowii*	CBS 385.80	*Senecio jacobaea* (Asteraceae)	UK	JF740244	JF740313	
*Leptosphaeria veronicae*	CBS 145.84	*Veronica chamaedryoides* (Scrophulariaceae)	Netherlands	JF740254	JF740320	
*Neoleptosphaeria rubefaciens*	CBS 387.80	*Tilia* (×) *europea* (Malvaceae)	Netherlands	JF740242	JF740311	
***Ochraceocephala foeniculi***	**Di3AF1 = CBS 145654**	***Foeniculum vulgare* (Apiaceae)**	**Italy**	**MN516753**	**MN516774**	**MN516743**
*Paraleptosphaeria dryadis*	CBS 643.86	*Dryas octopetala* (Rosaceae)	Switzerland	JF740213	GU301828	
*Paraleptosphaeria macrospora*	CBS 114198	*Rumex domesticus* (Chenopodiaceae)	Norway	JF740238	JF740305	
*Paraleptosphaeria nitschkei*	CBS 306.51	*Cirsium spinosissimum* (Asteraceae)	Switzerland	JF740239	JF740308	
*Paraleptosphaeria orobanches*	CBS 101638	*Epifagus virginiana* (Orobanchaceae)	USA	JF400230	JF740299	
*Paraleptosphaeria padi*	MFLU 15-2756	*Prunus padus* (Rosaceae)	Russia	KY554203	KY554198	KY554201
*Paraleptosphaeria praetermissa*	CBS 114591	*Rubus idaeus* (Rosaceae)	Sweden	JF740241	JF740310	
*Paraleptosphaeria rubi*	MFLUCC 14-0211	*Rubus* sp. (Rosaceae)	Italy	KT454726	KT454718	KT454733
*Paraleptosphaeria rumicis*	CBS 522.78	*Rumex alpinus* (Polygonaceae)	France	KF251144	KF251648	
*Plenodomus agnitus*	CBS 121.89	*Eupatorium cannabinum* (Asteraceae)	Netherlands	JF740194	JF740271	
*Plenodomus agnitus*	CBS 126584	*Eupatorium cannabinum* (Asteraceae)	Netherlands	JF740195	JF740272	
*Plenodomus artemisiae*	KUMCC 18-0151	*Artemisia* sp. (Asteraceae)	China	MK387920	MK387958	MK387928
*Plenodomus biglobosus*	CBS 119951	*Brassica rapa* (Brassicaceae)	Netherlands	JF740198	JF740274	JF740102
*Plenodomus biglobosus*	CBS 127249	*Brassica juncea* (Brassicaceae)	France	JF740199	JF740275	
*Plenodomus chrysanthemi*	CBS 539.63	*Chrysanthemum* sp. (Asteraceae)	Greece	JF740253	GU238151	GU238230
*Plenodomus collinsoniae*	CBS 120227	*Vitis coignetiae* (Vitaceae)	Japan	JF740200	JF740276	
*Plenodomus confertus*	CBS 375.64	*Anacyclus radiatus* (Asteraceae)	Spain	AF439459	JF740277	
*Plenodomus congestus*	CBS 244.64	*Erigeron canadensis* (Asteraceae)	Spain	AF439460	JF740278	
*Plenodomus deqinensis*	CGMCC 3.18221	soil	China	KY064027	KY064031	
*Plenodomus enteroleucus*	CBS 142.84	*Catalpa bignonioides* (Bignoniaceae)	Netherlands	JF740214	JF740287	
*Plenodomus enteroleucus*	CBS 831.84	*Triticum aestivum* (Poaceae)	Germany	JF740215	JF740288	
*Plenodomus fallaciosus*	CBS 414.62	*Satureia montana* (Lamiaceae)	France	JF740222	JF740292	
*Plenodomus guttulatus*	MFLU 15-1876	unidentified dead stem	Germany	KT454721	KT454713	KT454729
*Plenodomus hendersoniae*	CBS 113702	*Salix cinerea* (Salicaceae)	Sweden	JF740225	JF740295	
*Plenodomus hendersoniae*	CBS 139.78	*Pyrus malus* (Rosaceae)	Netherlands	JF740226	JF740296	
*Plenodomus hendersoniae*	LTO	*Salix appendiculata* (Salicaceae)	Austria	MF795790	MF795790	
*Plenodomus influorescens*	CBS 143.84	*Fraxinus excelsior* (Oleaceae)	Netherlands	JF740228	JF740297	
*Plenodomus influorescens*	PD 73/1382	*Lilium* sp. (Liliaceae)	Netherlands	JF740229	JF740298	
*Plenodomus libanotidis*	CBS 113795	*Seseli libanotis* (Apiaceae)	Sweden	JF740231	JF740300	
*Plenodomus lijiangensis*	KUMCC 18-0186	dead fern fronds	China	MK387921	MK387959	MK387929
*Plenodomus lindquistii*	CBS 386.80	*Helianthus annuus* (Asteraceae)	former Yugoslavia	JF740232	JF740301	
*Plenodomus lindquistii*	CBS 381.67	*Helianthus annuus* (Asteraceae)	Canada	JF740233	JF740302	
*Plenodomus lingam*	CBS 275.63	*Brassica* sp. (Brassicaceae)	UK	JF740234	JF740306	JF740103
*Plenodomus lingam*	CBS 260.94	*Brassica oleracea* (Brassicaceae)	Netherlands	JF740235	JF740307	
*Plenodomus lupini*	CBS 248.92	*Lupinus mutabilis* (Fabaceae)	Peru	JF740236	JF740303	
*Plenodomus pimpinellae*	CBS 101637	*Pimpinella anisum* (Apiaceae)	Israel	JF740240	JF740309	
*Plenodomus salviae*	MFLUCC 13-0219	*Salvia glutinosa* (Lamiaceae)	Italy	KT454725	KT454717	KT454732
*Plenodomus sinensis*	MFLU 17-0757	*Plukenetia volubilis* (Euphorbiaceae)	China	MF072722	MF072718	MF072720
*Plenodomus tracheiphilus*	CBS 551.93	*Citrus limonium* (Rutaceae)	Israel	JF740249	JF740317	JF740104
*Plenodomus tracheiphilus*	CBS 127250	*Citrus* sp. (Rutaceae)	Italy	JF740250	JF740318	
*Plenodomus visci*	CBS 122783	*Viscum album* (Viscaceae)	France	JF740256	EU754195	EU754096
*Plenodomus wasabiae*	CBS 120119	*Wasabia japonica* (Brassicaceae)	Taiwan	JF740257	JF740323	
*Plenodomus wasabiae*	CBS 120120	*Wasabia japonica* (Brassicaceae)	Taiwan	JF740258	JF740324	
*Pseudoleptosphaeria etheridgei*	CBS 125980	*Populus tremuloides* (Salicaceae)	Canada	JF740221	JF740291	
*Sphaerellopsis filum*	CBS 317.68	*Puccinia deschampsiae* uredinium, on *Deschampsia caespitosa*	Germany	KP170657	KP170725	
				
*Sphaerellopsis hakeae*	CPC 29566	*Hakea* sp. (Proteaceae)	Australia	KY173466	KY173555	
*Sphaerellopsis isthmospora*	KUN-HKAS 102225	Unidentified twig	China	MK387925	MK387963	MK387934
*Sphaerellopsis macroconidialis*	CBS 233.51	*Uromyces caryophylli* on *Dianthus caryophyllus*	Italy	KP170658	KP170726	
*Sphaerellopsis paraphysata*	CPC 21841	*Pennisetum* sp. (Poaceae)	Brazil	KP170662	KP170729	
*Subplenodomus apiicola*	CBS 285.72	Apium graveolens var. rapaceum (Apiaceae)	Germany	JF740196	GU238040	
*Subplenodomus drobnjacensis*	CBS 269.92	*Eustoma exaltatum* (Gentianaceae)	Netherlands	JF740211	JF740285	JF740100
*Subplenodomus valerianae*	CBS 630.68	*Valeriana phu* (Valerianaceae)	Netherlands	JF740251	GU238150	
*Subplenodomus violicola*	CBS 306.68	*Viola tricolor* (Violaceae)	Netherlands	FJ427083	GU238156	GU238231

Maximum likelihood (ML) analyses were performed with RAxML (Stamatakis 2006) as implemented in raxmlGUI 1.3 (Silvestro and Michalak 2012), using the ML + rapid bootstrap setting and the GTRGAMMA substitution model with 1000 bootstrap replicates.

Maximum parsimony (MP) bootstrap analyses were performed with PAUP v. 4.0a165 (Swofford 2002). All molecular characters were unordered and given equal weight; analyses were performed with gaps treated as missing data; the COLLAPSE command was set to MINBRLEN. MP bootstrap analyses were performed with 1000 replicates, using 5 rounds of random sequence addition and subsequent TBR branch swapping (MULTREES option in effect, steepest descent option not in effect) during each bootstrap replicate. In the Results and Discussion, bootstrap values below 70 % are considered low, between 70–90 % medium and above 90 % high.

### Pathogenicity test

To determine the ability of the representative isolate Di3A-F1 (CBS 145654) to cause disease symptoms, pathogenicity tests were conducted on 6-month-old plants of fennel grown in a growth chamber. Five plants for each of the three replicates were used. The inoculum, which consisted of a 6-mm-diameter mycelial plug from a 10-day-old culture on PDA, was inserted in four points for each crown and the wounds wrapped with Parafilm to prevent desiccation. Fennel plants inoculated with sterile PDA plugs served as a control. After inoculation, plants were covered with a plastic bag for 48 h and maintained at 25 ± 1 °C and 95% relative humidity (RH) under a 12 h fluorescent light/dark regime. Five days after inoculation the presence of a lesion was evaluated in each inoculation point. To fulfill Koch’s postulates, symptomatic tissues taken from the crown of each inoculated plant were plated on PDA and the identity of the fungal isolates was confirmed as described above.

### Cultivar susceptibility

To evaluate the susceptibility of six different cultivars of fennel to infection by the pathogen, one experiment was conducted on 1 to 2-month-old seedlings of fennel in a growth chamber. Eight plants for each of three replicates were used. The inoculum, which consisted of a 6-mm-diameter mycelial plug from a 10-day-old culture on PDA, was inserted at the crown of each plant and wrapped with Parafilm to prevent desiccation. Fennel plants inoculated with sterile PDA plugs served as a control. All the replicates were enclosed in plastic bags and maintained at 25 ± 1 °C and 95% relative humidity (RH) under a 12 h fluorescent light/dark regime in a growth chamber until the symptoms were observed. Plant mortality (PM), disease incidence (DI) and symptom severity (SS) were evaluated. Symptom severity was rated using a category scale from 0 to 5, where 0 = healthy plant; 1 = necrotic lesion on crown from 0.1 to 0.2 cm; 2 = from 0.3 to 1 cm; 3 = from 1.1 to 2 cm; 4 = from 2.1 to 3.5 cm; 5 = dead plant. The experiment was performed twice.

### Statistical analysis

Data about disease susceptibility of examined fennel cultivars from the repeated experiments were analysed by using the Statistica package software (v. 10; Statsoft Inc., Tulsa, OK, USA). The arithmetic means of PM, DI, and SS were calculated, averaging the values determined for the single replicates of each treatment. Percentage data concerning PM and DI were transformed into the arcsine (sin^–1^ square rootx) prior to analysis of variance (ANOVA), whereas SS values were not transformed. Initial analyses of PM and DI were performed by calculating F and P values associated to evaluate whether the effects of single factor (cultivar) and cultivar × trial interactions are significant. In the post hoc analyses, the corresponding mean values of PM and DI were subsequently separated by the Fisher’s least significant difference test (*P* = 0.05). Because ordinal scales were adopted for SS data calculation, different nonparametric approaches were used. Kendall’s coefficient of concordance (*W*) was calculated to assess whether the rankings of the SS scores among fennel cultivars are similar within each trial (cultivar × trial interactions). Since in the susceptibility experiment *W* was higher than 0.9, the SS scores were at first analysed by using Friedman’s nonparametric rank test, and subsequently followed by the all possible pairwise performed with the Wilcoxon signed-rank at *P* < 0.05. On the other hand, when only the cultivar effects were examined, the Kruskal-Wallis non parametric one-way test was preliminarily applied, calculating χ*^2^* and *P* value associated.

## Results

### Collection of samples and isolates

**Figure 1.**
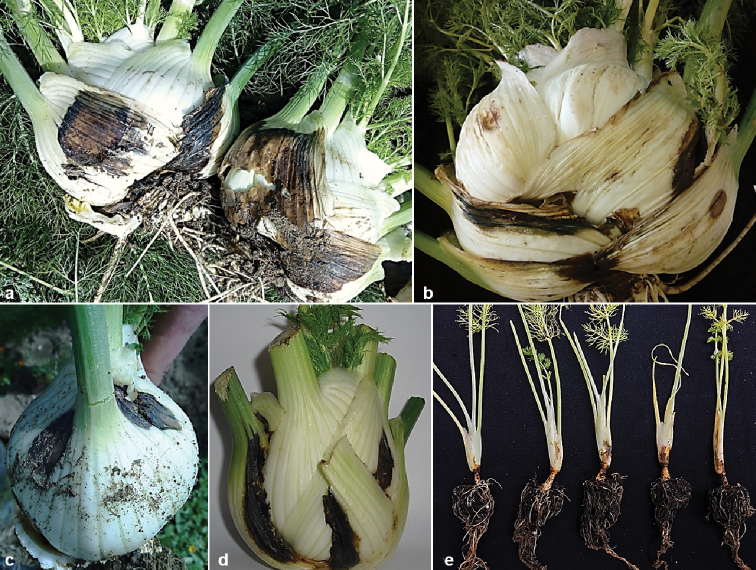
Symptoms caused by *Ochraceocephala foeniculi* on fennel plants. **a, b** Necrotic lesions and crown rot on ‘Narciso’ cultivar. **c, d** Necrotic lesions and crown rot on ‘Apollo’ cultivar. **e** Symptoms on artificially inoculated seedlings of ‘Pompeo’ cultivar.

Symptoms referable to infection (Fig. 1a, b) were detected in five commercial farms surveyed in eastern Sicily, Italy. The disease was observed on 3 different cultivars of fennel (4 to 6-month-old) in open fields. The symptoms consisted of depressed necrotic lesions formed near the soil line and affected crown, root, and stem. The lesion was first light brown with wet appearance, becoming dark brown to black with age and sometimes appearing dry. Under favourable conditions (high humidity), the lesion extended and the infection resulted in a crown and root rot. Fungal colonies representing the new fennel pathogen were consistently obtained from symptomatic tissues. A total of 32 single-spore isolates were collected (Table 3). Preliminary identity of the fungal isolates was based on cultural and morphological characteristics. Among these, 17 isolates were obtained from ‘Apollo’, 14 from ‘Narciso’, and one from ‘Aurelio’ cultivars.

### Sequencing

All strains of the new fennel pathogen sequenced had identical LSU, SSU, *RPB2*, *TEF1*, and *TUB2* sequences. Also all ITS sequences were identical, except for a single nucleotide polymorphism (A/G) towards the end of the ITS2 region. All sequences generated during this study were deposited at GenBank; for GenBank accession numbers, see Table 3.

### Phylogenetic analyses

**Figure 2.**
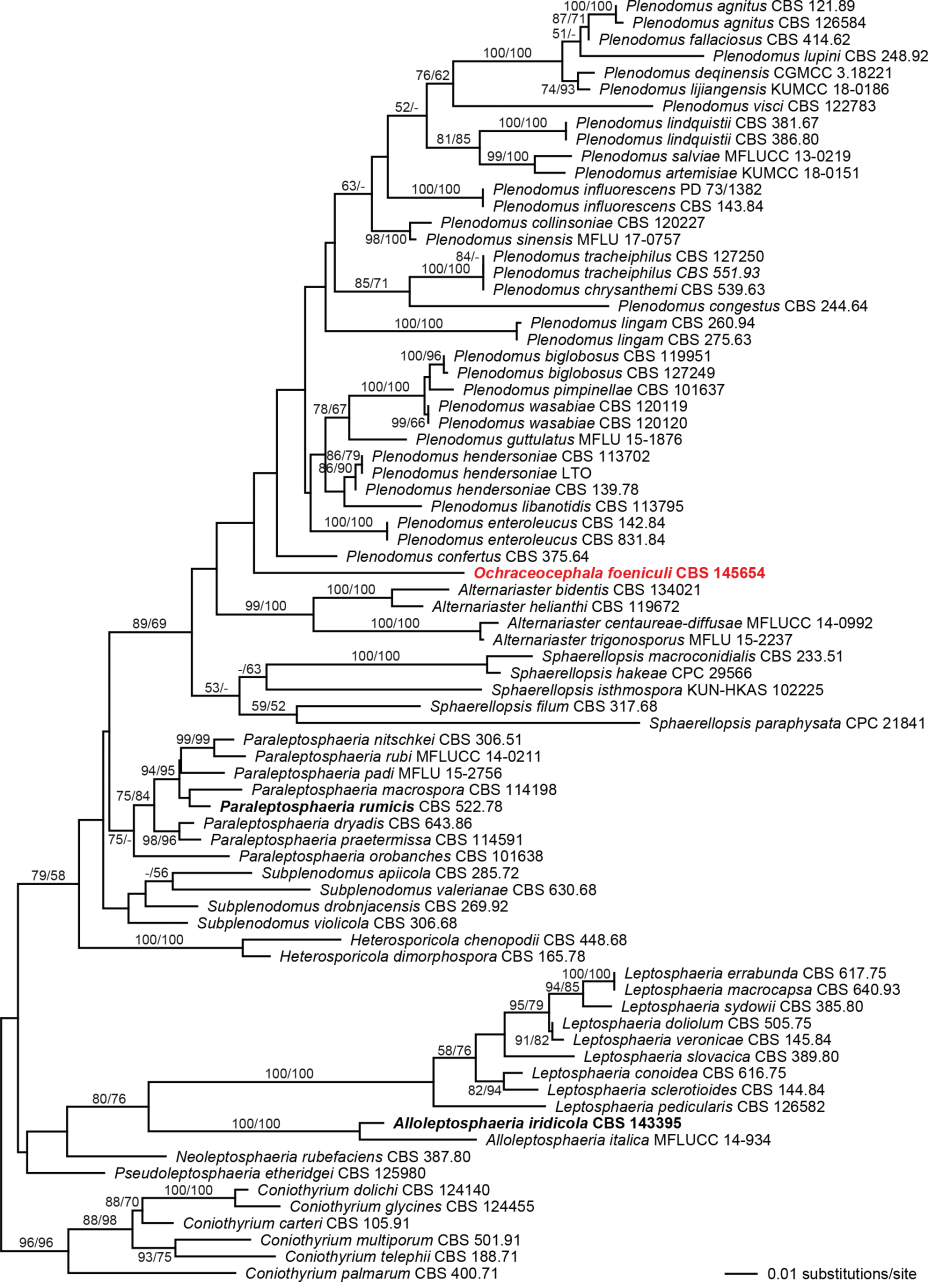
Phylogram of the best ML tree (–lnL = 14211.5558) revealed by RAxML from an analysis of the combined SSU-ITS-LSU matrix of selected Leptosphaeriaceae, showing the phylogenetic position of *Ochraceocephala foeniculi* (bold red). Taxa in bold black denote new combinations proposed here. ML and MP bootstrap support above 50% are given above or below the branches.

Of the 3312 characters included in the phylogenetic analyses, 294 were parsimony informative (222 from the ITS, 62 from the LSU, 10 from the SSU). The best ML tree (lnL = –14211.5558) revealed by RAxML is shown in Figure 2. In the phylogenetic tree, the Leptosphaeriaceae received high (96% ML and MP) support. Within Leptosphaeriaceae, most of the deeper nodes of the tree backbone received low to insignificant support. Highly supported genera include *Alloleptosphaeria*, *Heterosporicola*, *Leptosphaeria* (all three with maximum support) and *Alternariaster* (99% ML and 100% MP), while *Sphaerellopsis* received low (53%) and *Paraleptosphaeria* medium (75%) support only in the ML analyses, and *Plenodomus* and *Subplenodomus* were unsupported. *Subplenodomus iridicola* was not contained within the *Subplenodomus* clade, but sister species to *Alloleptosphaeria italica* with maximum support, and *Acicuseptoria rumicis* was embedded within the *Paraleptosphaeria* clade, indicating that they are generically misplaced. The new fennel pathogen was placed basal to the *Plenodomus* clade, however, without significant support. Although the new fennel pathogen is closely related to the genus *Plenodomus*, it is morphologically highly distinct. As no suitable described genus is available, a new genus is therefore established here.

### Taxonomy

#### Ochraceocephala

Taxon classificationFungiPleosporalesLeptosphaeriaceae

Voglmayr & Aiello gen. nov.

0B00DF28-64E0-594C-BDF0-DC6878ABD45B

833933

##### Etymology.

referring to the ochraceous conidial capitula of the type species.

Conidiophores erect, variable in shape and branching, from unbranched, loosely to densely branched up to several times; branching commonly irregularly verticillate. Phialides arising singly or in irregular whorls, cylindrical, lageniform or ampulliform, producing basipetal conidial chains. Conidia in chains, unicellular, thick-walled.

##### Type species.

*Ochraceocephala foeniculi* Voglmayr & Aiello.

##### Notes.

*Ochraceocephala* is phylogenetically closely related to *Plenodomus*, from which it deviates substantially in morphology. *Plenodomus* species are characterised by pycnidial phoma-like asexual morphs, and while in two *Plenodomus* species (*P. chrysanthemi*, *P. tracheiphilus*) simple hyphomycetous, phialophora-like synanamorphs have been recorded (Boerema et al. 1994), these are very different from the complex conidiophores of the present fennel pathogen. These morphological differences, the lack of a suitable genus within Leptosphaeriaceae and its phylogenetic position therefore warrants the establishment of a new genus.

#### Ochraceocephala foeniculi

Taxon classificationFungiPleosporalesLeptosphaeriaceae

Voglmayr & Aiello sp. nov.

B4B33652-7F10-53D8-A3BF-C15E6F892212

833934

Figure 3

##### Etymology.

referring to its host genus, *Foeniculum* (Apiaceae).

Colonies fast-growing, at room temperature (22 ± 1 °C) on CMD reaching 80 mm after 7 d; on MEA 38 mm after 7 d; with dull white to cream surface, upon conidiation becoming beige to olive yellow from the centre, reverse cream with greyish to dark brown centre; cottony, with abundant surface mycelium; sporulation abundant on aerial hyphae. Aerial hyphae hyaline, 2–6 µm wide. Conidiophores hyaline, produced terminally or laterally on aerial hyphae, variable in shape and branching, unbranched, loosely or densely branched up to two times; branching commonly irregularly verticillate. Phialides arising singly or in whorls of 2–5, (3.8–)5.8–13.5(–21.0) × (2.5–)3.0–4.3(–5.5) µm (*n* = 100), cylindrical, lageniform or ampulliform, often with a distinct collarette, producing basipetal conidial chains; polyphialides rarely present. Conidia (3.2–)3.5–6.0(–8.5) × (2.5–)3.0–4.2(–6.0) µm, l/w (1.0–)1.1–1.5(–2.1) (*n* = 155), hyaline to yellowish, in masses sand to olive yellow, smooth, mostly globose to subglobose, rarely broadly ellipsoid to pip-shaped, thick-walled.

**Figure 3.**
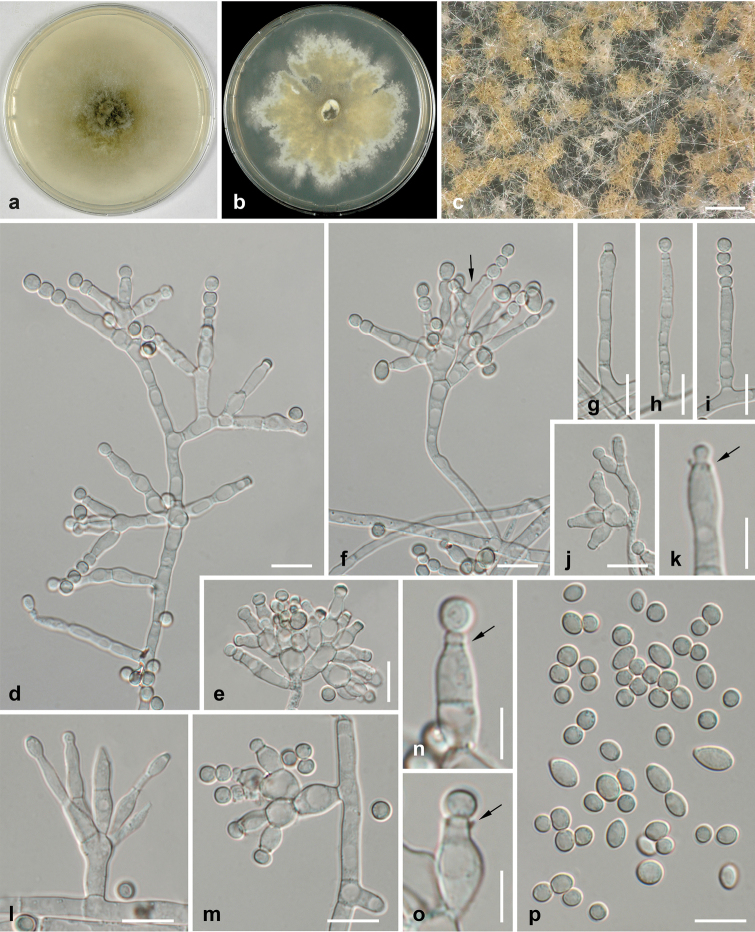
*Ochraceocephala foeniculi*, holotype **a** culture on CMD (7d, 22 °C) **b** culture on MEA (21d, 22 °C) **c** conidiophores on aerial hyphae producing yellowish brown conidial masses in chains **d–j, l, m** unbranched (**g–i**) and verticillately branched (**d–f, j, l, m**) conidiophores (MEA, 21d, 22 °C) with phialides; in **f** with polyphialide (arrow) **k, n, o** phialides with collarettes (arrows) and young conidia **p** conidia. All microscopic preparations from MEA (21d, 22 °C) and mounted in water. Scale bars: 200 µm (**c**); 10 µm (**d–j, l**, **m**, **p**); 5 µm (**k**, **n**, **o**).

##### Distribution.

Italy (Sicily).

##### Host and substrate.

Pathogenic on crown, roots and stems of living *Foeniculum vulgare*.

##### Holotype.

Italy, Sicily, Catania province, Adrano, May 2017 (WU 40034); ex-holotype culture CBS 145654; ex holotype sequences MN516753 (ITS), MN516774 (LSU), MN516743 (SSU), MN520145 (*RPB2*), MN520149 (*TEF1*), MN520147 (*TUB2*).

#### Alloleptosphaeria iridicola

Taxon classificationFungiPleosporalesLeptosphaeriaceae

(Crous & Denman) Voglmayr comb. nov.

CA84974D-1BCD-5F6E-9D3A-876A4A40A4CF

833935

##### Basionym.

*Subplenodomus iridicola* Crous & Denman, in Crous, Schumacher, Wingfield, Akulov, Denman, Roux, Braun, Burgess, Carnegie, Váczy, Guatimosim, Schwartsburd, Barreto, Hernández-Restrepo, Lombard & Groenewald, Fungal Systematics and Evolution 1: 207. 2018.

##### Notes.

In the phylogenetic analyses (Fig. 2) *Subplenodomus iridicola* is placed remote from the other species of *Subplenodomus*, but is sister species to *Alloleptosphaeria italica* with maximum support; *S. iridicola* is therefore transferred to the genus *Alloleptosphaeria*.

#### Paraleptosphaeria rumicis

Taxon classificationFungiPleosporalesLeptosphaeriaceae

(Quaedvl., Verkley & Crous) Voglmayr comb. nov.

AE4F5402-1ED1-537A-AB77-18ED8DA180BD

833936

##### Basionym.

*Acicuseptoria rumicis* Quaedvl., Verkley & Crous, Stud. Mycol. 75: 376 (2013).

##### Notes.

The monotypic genus *Acicuseptoria* was described by Quaedvlieg et al. (2013) as a segregate of the polyphyletic genus *Septoria*, and it was characterised by brown, globose pycnidia with conidiophores reduced to ampulliform conidiogenous cells bearing acicular, hyaline, euseptate conidia. However, its position within the Leptosphaeriaceae remained undetermined as no other representatives of the family were included in their phylogenetic tree (Quaedvlieg et al. 2013: fig. 2). In our phylogenetic analyses (Fig. 2), *Acicuseptoria rumicis* is embedded within the genus *Paraleptosphaeria* and placed in a highly supported subclade that also contains the generic type, *P. nitschkei*. *Acicuseptoria rumicis* is therefore transferred to the genus *Paraleptosphaeria*.

### Pathogenicity test

The representative isolate (CBS 145654) was pathogenic to fennel plants, and produced symptoms similar to those observed in open field after five days (Fig. 1e). The pathogen was re-isolated from the artificially inoculated plants, and identified as previously described. No symptoms were observed on control plants.

### Cultivar susceptibility

In the experiments on fennel susceptibility there was always a significant effect of the cultivar on all disease parameters (PM, DI and SS) of pathogen infections (*p* < 0.0001). Otherwise, a not significant cultivar × trial effect (*p* > 0.56) was observed for parametric variables (PM and DI) in this repeated experiment (Table 5). Besides, Kendall’s coefficient of concordance was 0.96 for SS data, thus indicating very high concordance between the two trials (Table 5). Therefore, the two trials were combined.

**Table 5.** ANOVA effects of cultivar and cultivar × trial interactions on plant mortality, disease incidence and severity of symptoms caused by *Ochraceocephala foeniculi* on inoculated young fennel plants.

**Model effect**	**Parameter**								
**Factor(s)**	**Plant mortality (PM)** ^1^			**Disease incidence (DI)** ^1^			**Symptom severity (SS)** ^2^		
	**df**	***F***	***P* value**	**df**	***F***	***P* value**	χ***^2^***	***W***	***P* value**
** Cultivar **	5	70.6286	< 0.0001	5	33.659	< 0.0001	89.2051	…	< 0.0001
** Cultivar × trial **	5	0.1273	0.98475^ns^	5	0.789^ns^	0.56797^ns^	…	0.95873	0.0003

^1^*F* test of fixed effects, df = degrees of freedom, and *P* value associated to *F*; ns = not significant. ^2^ The χ*^2^* value for Kruskal-Wallis one-way analysis of variance test (cultivar) and Friedman two-way analysis of variance (cultivar × trial), respectively; *W* = Kendall’s coefficient of concordance between repeated trials in the experiment.

Regarding susceptibility of fennel to this phytopathogenic fungus, a great variability was detected among the tested cultivars eight days after inoculation. Comprehensively, cultivar ‘Narciso’ was the most susceptible since all disease parameters and its PM value were significantly the highest among the tested cultivars. ‘Apollo’ was also highly susceptible to infection by the new fennel pathogen, significantly differing only in a slightly lower PM value. ‘Pompeo’ displayed PM and DI values similar to those recorded for ‘Apollo’, but its SS score was significantly lower than in the former (Table 6). In decreasing order of susceptibility, ‘Aurelio’ did not significantly differ from ‘Pompeo’ for DI and SS values, but its PM caused by the fennel pathogen was strongly reduced. No dead seedlings (PM = 0) were recorded for both ‘Archimede’ and ‘Pegaso’, that significantly differed for DI and SS from the other remaining cultivars. Altogether, ‘Pegaso’ was the least susceptible cultivar to fungal infection since it showed the lowest values of disease severity.

**Table 6.** Compared susceptibility to crown and root rot infections of six commercial fennel cultivars.

**Cultivar**	**Plant mortality (PM)** ^1^	**Disease incidence (DI)** ^1^	**Symptom severity (SS)** ^2^
‘**Narciso**’	72.92 ± 2.08 a	100 a	4.15 ± 0.10 a
‘**Apollo**’	58.33 ± 4.17 b	100 a	4.33 ± 0.17 a
‘**Pompeo**’	45.83 ± 4.17 b	100 a	3.37 ± 0.13 b
‘**Aurelio**’	10.42 ± 2.08 c	100 a	2.56 ± 0.06 b
‘**Archimede**’	0.00 d	83.33 ± 4.17 b	1.94 ± 0.10 c
‘**Pegaso**’	0.00 d	77.08 ± 2.08 b	1.48 ± 0.10 d

^1^Data derived from repeated experiment. Standard error of the mean = SEM, means are from 24 fennel young plants. Arithmetic means are presented although analysis was performed on angular transformed values. Means followed by different letters within the column are significantly different according to Fisher's least significance differences test (α = 0.05). ^2^ Differences among SS (0-to-5 scale) data for each treatment were analysed with Friedman two-way analysis of variance by mean rank scores (*P* < 0.001) followed by all pairwise multiple comparison with Wilcoxon.

## Discussion

In the present study, 32 fungal isolates were recovered from symptomatic fennel plants in Sicily over a 2-year period. Disease symptoms were observed in three farms, and included necrotic lesions and crown and root rot on three different cultivars. The fungal species obtained from symptomatic tissues was identified based on morphological characters and molecular phylogenetic analyses of an ITS-LSU-SSU rDNA matrix, resulting in the description of the fennel pathogen as a new genus and species, *Ochraceocephala foeniculi*.

In the phylogenetic analyses, *O. foeniculi* was revealed as sister group of *Plenodomus*; however, without significant support (Fig. 2). As commonly observed with ITS-LSU-SSU rDNA data, support of many backbone nodes is low or absent, and additional protein-coding markers like *RPB2*, *TEF1* and *TUB2* are necessary for an improved phylogenetic resolution of genera and families in Pleosporales (Voglmayr and Jaklitsch 2017; Jaklitsch et al. 2018). Although we sequenced *RPB2*, *TEF1*, and *TUB2* for *O. foeniculi*, it was currently not feasible to perform multi-gene analyses due to insufficient sequence data for most species of Leptosphaeriaceae, in particular for *Plenodomus*. However, we consider the phylogenetic and morphological evidence conclusive for establishing the new genus *Ochraceocephala*. Also the generic transfer of *Subplenodomus iridicola* to *Alloleptosphaeria* is well substantiated, considering its highly supported phylogenetic position as sister species of *Alloleptosphaeria italica*, remote from the generic type (*S. violicola*) and other species of *Subplenodomus* (Fig. 2). In the phylogenetic analyses of the LSU rDNA matrix of Crous et al. (2018: fig. 1), only few taxa of Leptosphaeriaceae were included, and the phylogenetic position of *S. iridicola* remained inconclusive due to low resolution; however, also in their analyses it was placed remote from the generic type, *S. violicola*. In addition, they did not include its closest relative, *Alloleptosphaeria italica*, although it was mentioned as the closest match of an ITS BLAST search (Crous et al. 2018). No asexual morph is known for *A. italica* (Dayarathne et al. 2015), but the ascomata, asci and ascospores of *A. iridicola* and *A. italica* share many traits. Our phylogenetic analyses also showed that *Acicuseptoria rumicis* should be included within *Paraleptosphaeria* (Fig. 2). Although it was correctly placed within Leptosphaeriaceae by Quaedvlieg et al. (2013), its position within the family remained undetermined as no other representatives of the family were included in their phylogenetic analyses. As for most other species of *Paraleptosphaeria* no asexual morphs are known, no comprehensive morphological comparison can currently be made with *P. rumicis*.

Within Leptosphaeriaceae, *O. foeniculi* is remarkable and unique by its complex hyphomycetous asexual morph composed of branched conidiophores with phialidic conidiation and conidia produced in basipetal chains. Asexual morphs in Leptosphaeriaceae are typically coelomycetous and phoma-like, which is also the case in the closest relative of *Ochraceocephala*, *Plenodomus* (Gruyter et al. 2013). Another genus of Leptosphaeriaceae with a hyphomycetous asexual morph is *Alternariaster*, which, however, differs significantly by tretic condiogenous cells forming large, brown, septate conidia not produced in chains (Simmons 2007; Alves et al. 2013). Therefore, the unique morphology in combination with an isolated phylogenetic position within Leptosphaeriaceae warrant the establishment of a new genus.

Other fungal species belonging to Leptosphaeriaceae, as well as the closely related Didymellaceae (Odstrčilová et al. 2002; Shaker and Alhamadany 2015) have been reported worldwide in fennel crops. In Italy, crown rot of fennel caused by *Didymella glomerata* (syn. *Phoma glomerata*) was recorded from southern Italy (Lahoz et al. 2007). As confirmed in the pathogenicity tests, *O. foeniculi* caused symptoms on artificially inoculated plants of the same cultivar and, moreover, also on different fennel cultivars that showed some variability in disease susceptibility. To this regard, it is noteworthy that this study also represents a preliminary evaluation of fennel germplasm according to their susceptibility to this new disease. Although these data should be confirmed by additional investigations, this study might provide very useful information for local farmers and technicians. The determination of the extent of susceptibility to *O. foeniculi* is a starting point for evaluating the tolerance of commercial fennel cultivars to this disease under different agronomic and phytosanitary conditions.

On the basis of the disease incidence and severity observed in the field, we believe that this disease represents a serious threat to fennel crop in Sicily and may become a major problem also to other areas of fennel production if accidentally introduced. Moreover, infected soil could represent an inoculum source for this fungus. Further studies are needed to examine the life cycle of *O. foeniculi* and to ascertain the cardinal temperatures of the fungus for successful infection since this pathogen is well established in this representative fennel production area. This information is required for the setup and timing of sustainable approaches for soil disinfection, including solarization and/or fumigation at low rates, to reduce the level of the primary inoculum in the soil and hence the disease amount, like successfully applied for other soilborne plant pathogens (Vitale et al. 2013; Aiello et al. 2018).

Although not always conclusive, soil disinfestation and host resistance can be considered environmentally friendly means to be included within integrated pest management (IPM) strategies against crown rot caused by *O. foeniculi* in order to minimize the number and intensity of fungicide applications.

## Supplementary Material

XML Treatment for Ochraceocephala

XML Treatment for Ochraceocephala foeniculi

XML Treatment for Alloleptosphaeria iridicola

XML Treatment for Paraleptosphaeria rumicis
